# Effect of skill drills on neonatal ventilation performance in a simulated setting- observation study in Nepal

**DOI:** 10.1186/s12887-019-1723-0

**Published:** 2019-10-28

**Authors:** Rejina Gurung, Abhishek Gurung, Avinash K. Sunny, Omkar Basnet, Shree Krishna Shrestha, Øystein Herwig Gomo, Helge Myklebust, Sakina Girnary, Ashish KC

**Affiliations:** 1 Golden Community, Jawgal-11, Lalitpur, Nepal; 2 Pokhara Academy of Health Sciences, Ramghat 10, Pokhara, Nepal; 3 Laerdal Medical / Laerdal Global Health, Stavanger, Norway; 4 Department of Women’s and Children’s Health, Uppsala University, Dag Hammarskjölds väg 14B, floor 1, Uppsala, Sweden

**Keywords:** Neonatal resuscitation, Skill drills, Feedback, Simulated setting, Quality improvement

## Abstract

**Aim:**

Maintaining neonatal resuscitation skills among health workers in low resource settings will require continuous quality improvement efforts. We aimed to evaluate the effect of skill drills and feedback on neonatal resuscitation and the optimal number of skill drills required to maintain the ventilation skill in a simulated setting.

**Methods:**

An observational study was conducted for a period of 3 months in a referral hospital of Nepal. Sixty nursing staffs were trained on Helping Babies Breathe (HBB) 2.0 and daily skill drills using a high-fidelity manikin. The high-fidelity manikin had different clinical case scenarios and provided feedback as “well done” or “improvement required” based on the ventilation performance. Adequate ventilation was defined as bag-and-mask ventilation at the rate of 40–60 breaths per minute. The effective ventilation was defined as adequate ventilation with a “well done” feedback. We assessed the correlation of number skill drills and clinical case scenario with adequate ventilation rate using pearson’s correlation. We assessed the correlation of number of skill dills performed by each participant with effective ventilation using Mann Whitney test.

**Results:**

Among the total of 60 nursing staffs, all of them were competent with an average score of 12.73 ± 1.09 out of 14 (*p* < 0.001) on bag-and-mask ventilation skill checklist. Among the trained staff, 47 staffs participated in daily skill drills who performed a total of 331 skill drills and 68.9% of the ventilations were done adequately. Among the 47 nursing staffs who performed the skill drills, 228 (68.9%) drills were conducted at a ventilation rate of 40–60 breathes per minute. There was no correlation of the adequate ventilation with skill drill category (*p* = 0.88) and the level of skill performed (*p* = 0.28). Out of 47 participants performing the skill drills, 74.5% of them had done effective ventilation with a mean average of 8 skill drills (SD ± 4.78) (*p*-value- 0.032).

**Conclusion:**

In a simulated setting, participants who had an average skill drill of 8 in 3 months had effective ventilation. We demonstrated optimal skill drill sessions for maintain the neonatal resuscitation competency. Further evaluation will be required to validate the findings in a scale up setting.

## What is known of this subject

- Quality improvement interventions include training, reminder, audit and feedback, improve in skill and maintain the neonatal resuscitation skills
- There is a subsequent decay in the neonatal resuscitation skills if continuous refresher trainings are not provided
- Neonatal resuscitation training and retraining require resources

## What is new from this study

- Self-training with a high-fidelity, feedback simulator can maintain the neonatal resuscitation skills
- For effective ventilation, the ventilation rate should be maintained along with the automated and objective feedback from the high-fidelity simulator
- At least 8 skill drills are optimal to maintain the neonatal resuscitation skill for a period of 3 months

## Background

Estimated 5.5 million deaths take place every year after 28 weeks of pregnancy within the neonatal period. Of these deaths, 1.8 million deaths pertain to intrapartum [1, 2]. The Lancet Global Health Commission on High Quality Health Systems in the Sustainable Development Goal Era estimates that almost two-third of these deaths are associated with poor quality of care [3]. Every Newborn Action Plan-2014 aims to reduce the preventable stillbirth and neonatal death by 2030 in all income settings [4]. Reducing preventable death requires high quality care and systems for managing mothers and newborns. Systematic review has shown that neonatal resuscitation, if delivered in a high-quality standard, can reduce intrapartum related death by 30% [5]. Effective scale up of neonatal resuscitation program is required to maintain the effect of the intervention on intrapartum related death [6, 7]. However, there are barriers at different levels of governance (macro-, meso- and micro level) for an effective implementation of neonatal resuscitation [7]. Different quality improvement (QI) interventions are recommended at different levels of governance to overcome the barriers for effective implementation [8, 9]. The QI interventions at system level include governance and financing; at hospital level involves setting accountability for improving care, and at health workers level comprise training, reminders, audit and dissemination of the guideline [9].

American Academy of Pediatrics developed a pictorial based neonatal resuscitation program with training package called Helping Babies Breathe (HBB) for middle- and low-income settings in 2010 [10]. The objective of the program was to improve health worker’s performance in simulated as well as clinical settings [11]. Evaluation of HBB program has shown improvements in the health workers’ performance as well as mortality outcomes in low- and middle-income settings [12–14]. A systematic review of HBB implementation on birth outcomes showed 34 and 30% reduction in intrapartum stillbirth (RR-0.66; CI 95% 0.52–0.85) and first day mortality (RR-0.70; CI 95% 0.51–0.98), respectively [15].

Different quality improvement interventions- training, reminders and audit & feedback have been used to implement HBB program [16]. In a rural hospital in Tanzania, training and retraining was used as QI interventions for improving health worker’s neonatal resuscitation performance in a simulated setting [17]. In a multi-country study conducted in India and Kenya, daily skill drills and reminders were used as QI interventions for maintaining neonatal resuscitation skills [18]. In a tertiary hospital study in Nepal, audit and feedback was used as QI interventions for reducing perinatal mortality using HBB [13].

Despite the description of the QI interventions in HBB program, there are evidence gap on QI interventions that improve and maintain neonatal resuscitation performance in simulated settings [19–21]. One of the most recommended QI interventions in resuscitation is daily skill drills [22]. Barriers exist in implementation of daily skill drills in clinics for the health workers [23].

We aimed to evaluate the effect of skill drills and feedback on neonatal resuscitation performance and optimal skill drills required to maintain the ventilation skills in a simulated setting.

## Methods

This study is reported as per strengthening the reporting of observational studies in epidemiology (STROBE checklist) [24].

### Study design

This is an observational cohort study.

### Study Setting

We conducted this study in a referral level hospital of Nepal- Pokhara Academy of Health Sciences, located in western Nepal. The 250 bedded hospital has an annual delivery rate of 8560 with stillbirth rate of 19 per 1000 births and pre-discharge neonatal mortality of 15 per 1000 live births [25].

The hospital had four units for managing mothers and newborns.

1. Labor unit with two delivery beds were managed by obstetricians and nurses. The normal and complicated vaginal delivery took place in the labor unit. The labor unit had 15 nursing staffs.
2. Operating theatre was managed by obstetricians and surgeons, where caesarean section took place. There were two operating tables. There were total of 15 nursing staffs.
3. Postnatal unit managed by obstetricians and nurses. The mothers and newborns were kept in observation following low risk delivery. There were 8 beds with a total of 15 nursing staffs.
4. Neonatal Care Unit was managed by pediatricians and nurses where the sick newborns were kept for treatment. There were 8 radiant warmers, 2 CPAP machine and one phototherapy machine. There were total of 15 nursing staff.

The study was conducted from 15 July to 15 October 2018.

#### Sample size

We included all the nursing staff working at the maternal and newborn care units.

#### Participants

The nurses working in all four units were eligible as they have been working to provide care for mothers and babies at the time of birth and emergency condition. An orientation meeting was conducted with the nursing department on the objective of the study and nurses were consented. A total of 60 participants were enrolled in the study.

#### Interventions

The participants were given one-day training on HBB second edition (2.0) package [26]. The HBB 2.0 followed the International Liaison Committee on Resuscitation (ILCOR) 2015 updated guideline for management of the non-breathing baby’s basic neonatal resuscitation processes with the cord intact and less use of suctioning unless warranted [27]. HBB 2.0 is a skill-based training with practice on a manikin. The manikin (NeoNatalie) is a synthetic baby doll which is water filled and weighs 2.5 kg [28]. It has a plastic-foil lung in the chest which shows chest rise when ventilations are provided. The HBB training package also included upright bag-and-mask ventilation, reusable suction in the shape of a penguin, two cord ties, two sets of towels, a baby cap and an umbilical cord in the set. The training was organized in a batch of 20, arranged in a group of four in each table. Each table had two manikins such that the steps on neonatal resuscitation could be practiced in a pair for at least 40 times during the training session. On the first day, the standard training on HBB 2.0 was provided.

On the second day, the high-fidelity manikin (NeoNatalie Live) which simulate different clinical scenario was introduced to the training participants. NeoNatalie Live was developed to give a more life-like realism of the challenges that often occur in the resuscitation of newborn babies, to simulate a realistic lung- and heart rate development after birth, and to measure and help improve the skills of personnel performing newborn resuscitation [28].

This high-fidelity manikin had sensors that measure resuscitation skills in the form of head positioning for keeping the airway open, mask seal, air pressure given to the lungs, ventilation rate, and continuity of ventilation. After a sufficient time of adequate ventilation (40–60 breathe per minute), the manikin cried as a signal of spontaneous breathing. The manikin had wireless connectivity to a tablet-based application (app) from which three patient cases (skill levels) could be selected, including different initial heart rates, as well as the condition of the lungs. The levels were categorized as Easy, Medium and Hard. The ‘Easy’ level had a normal heart rate and open lungs. The ‘Medium’ level had a low heart rate and open lungs. The ‘Hard’ level had low heart rate with closed lungs. Immediately after each resuscitation practice, the app provided feedback to the participants on how the resuscitation was performed and how to improve next time. If the app provided feedback as “well done” then the participant had done the clinical case scenario as per the ventilation step. If not, the app provided feedback on the ventilation step which required improvement. The app also recorded the names of the participants and created a participant ID linked to the resuscitation practice. All results were automatically uploaded to a cloud database (Microsoft Azure).

The ventilation practice was performed with a new and improved resuscitator for newborns (the Laerdal Upright w/PEEP bag and mask). Equipped with a novel PEEP-valve, it adds a positive end-expiratory pressure in the lungs of newborns. The ergonomically improved upright design and new mask that makes it easier to maintain mask seal (Additional file 1).

Each participant in the different maternal and newborn care units were instructed to practice the neonatal resuscitation on the manikin before they started their clinical work in each unit every day. Nurses registered their name in the app and practiced skill drills on the manikin during the study period.

#### Data collection

The evaluation of HBB 2.0 was done using a knowledge-based questionnaire (pre and post), a skill-based checklist and clinical case scenarios (Additional file 2). The questionnaire for knowledge assessment had 17 multiple choice questions and 7 skill-based checklists for bag-and-mask ventilation evaluation. Two clinical scenario-based questionnaires - Objective Structured Clinical Evaluation (OSCE) A (13 steps) and OSCE B (23 steps) from the standard HBB evaluation package were used. The background information of the study participants such as age, education, experience in delivery care and resuscitation was assessed. Evaluation during the training was done in a paper-based format.

The level of skill drill for a particular day was randomly selected by an independent research officer and provided to the study participants. The number of skill drills performed by each participant was manually recorded by the research officer.

The app recorded information on study participants, level of skill drill performed and performance feedback following completion of each drill. Four dedicated research officers were assigned to observe the activities.

#### Quantitative variables

##### Dependent variables

Adequate ventilation-Ventilation at the rate of 40–60 breathe per minute,

Effective ventilation- Adequate ventilation with a “well done” feedback in 75% of the skill drill attempts,

##### Independent variable

Number of skill drills conducted,

The level of skill drill: easy, medium and hard clinical case scenario,

##### Background variables

The demographic characteristics of the study participants (age, education, experience in delivery and neonatal resuscitation)

Knowledge before and after the training

Skill and clinical scenario competency following the training (OSCE A and B)

#### Statistical methods

For comparison of knowledge of the study participants, a paired t-test was used, and mean difference was calculated. For skill competency of the study participants following training, one sample t-test was used, and mean difference was calculated. The number of skill drills was categorized into 1–5, 6–10 and ≥ 11 sessions. We assessed the correlation of number skill drills and clinical case scenario (level of skill-easy, medium and hard) with adequate ventilation rate. For this, pearson’s correlation was used to analyze the correlation between each category of skill drills and level of skill with adequate ventilation rate (40–60 breaths per minute) on each drill. We assessed the correlation of number of skill dills performed by each participant with effective ventilation (75% of the attempt having 40–60 breathe ventilation per minute with well-done feedback). For this, we used a non-parametric test (Mann Whitney test) which provided the mean (with standard deviation) and median (with inter-quartile range) for effective and ineffective ventilation.

#### Ethical approval

The study was approved by the Institutional Review Committee of the hospital as well as Ethical Review Board of Nepal Health Research Council (reg. 95–2018).

## Result

A total of 60 participants were eligible and enrolled in the study. Among them, 30 (50%) were staff nurses (Proficiency Certificate Level in nursing), 12 (20%) were auxiliary nurses (Auxiliary Nurse Midwife) and 18 (30%) were nurses with an academic qualification of bachelor’s and above. Regarding attending births per month in average, 5 (8.3%) had attended 1–5 births per month, 5 (8.3%) had attended 6–15 births per month and 17 (28.3%) had attended more than 16 births per month while 33 (55%) didn’t attend any births at all. For resuscitation, 62% of them had used bag-and-mask. Among the total staffs, 21.7% did not conduct any skill drills, 38.3% conducted 1–5 drills, 20.0% conducted 6–10 drills and 20.0% conducted 11 or more drills (Table 1).

**Table 1:** Demographics of the participants (*n* = 60)

Variables	N (%)
Education	
Auxiliary nurse	12 (20.0)
Staff Nurse	30 (50.0)
Bachelor nursing and above	18 (30.0)
Births attended/per month in average	
None	33 (55.0)
1 to 5	5 (8.3)
6 to 15	5 (8.3)
16 to 25	5 (8.3)
more than 25	12 (20)
Used a bag and mask to resuscitate a baby	
Yes	37 (61.7)
No	23 (38.3)
Drills category	
No sessions	13 (21.7)
1–5 sessions	23 (38.3)
6–10 sessions	12 (20.0)
≥ 11 sessions	12 (20.0)

There was an improvement in knowledge on HBB 2.0 by 1.77 (95% CI: 1.24–2.30) times among the participants. All participants scored more than 80% in the bag-and-mask skill check; average score of 12.73 out of 14 (*p* < 0.001). In the OSCE A and B, the post-training score was more than 80% of the provided checklist. In the ventilation graph, the average ventilation rate among the 60 participants was 40.55 ± 2.66 (Table 2).

**Table 2:** Competency before and after training among the nurses on Helping Babies Breathe 2.0 (*n* = 60)

		Mean ± SD	95% CI	*P* value
Total knowledge	Pre-test	15.62 ± 1.92	1.24–2.30	< 0.001^a^
	Post-test	17.38 ± 1.04		
OSCE A	Full score = 13	11.58 ± 0.70	11.40–11.76	< 0.001^b^
Bag and Mask ventilation	Full score = 14	12.73 ± 1.09	12.45–13.01	< 0.001^b^
OSCE B	Full score = 23	21.72 ± 1.59	21.31–22.31	< 0.001^b^
Ventilation Graph	Ventilation rate	40.55 ± 2.66	39.86–41.24	< 0.001^b^

^a^Paired sampled t-test, ^b^One Sample t-test

**Fig. 1:**
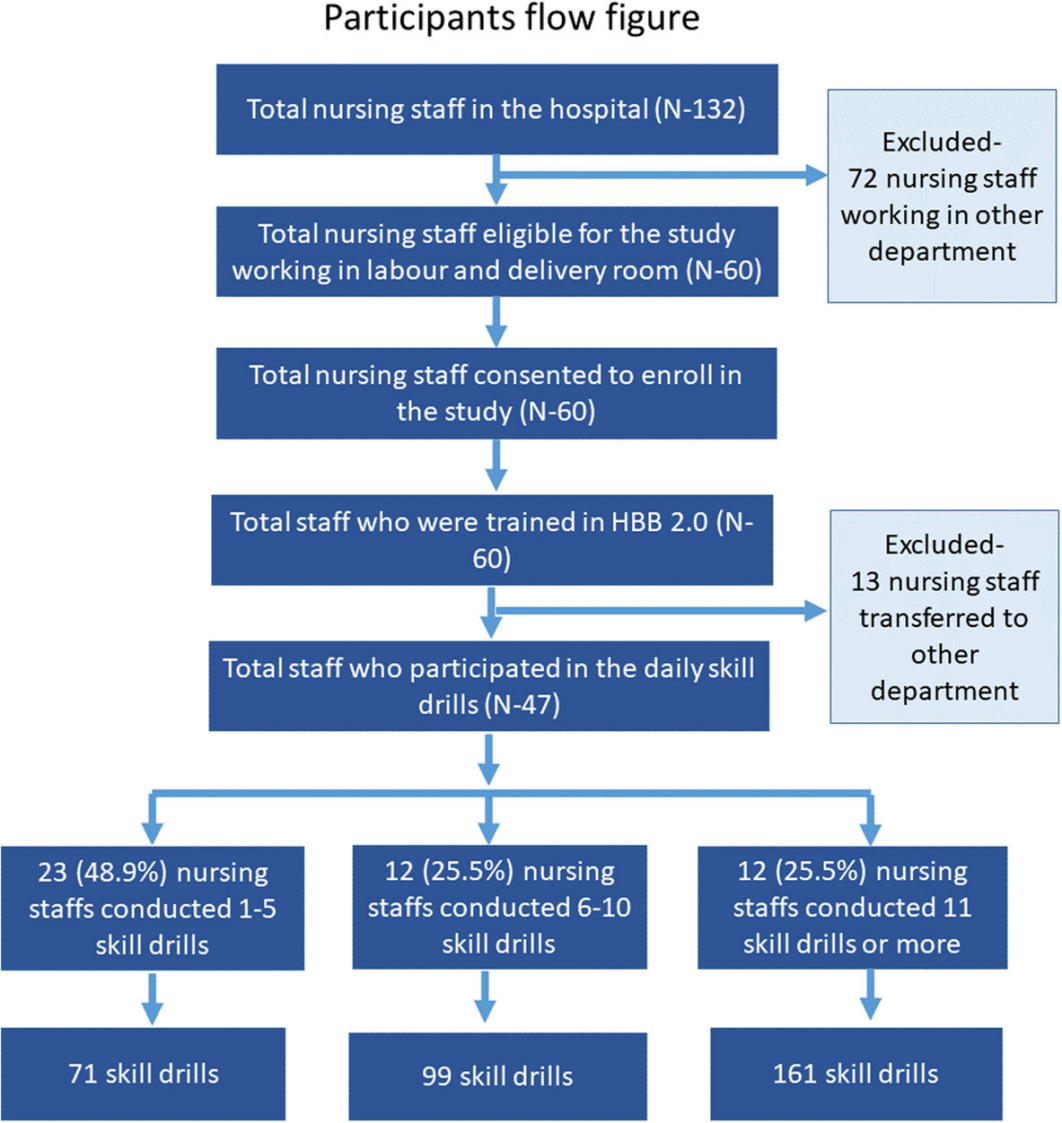
Study participant flow

Among the total participants, 47 participated in the skill drills and a total of 331 drills were conducted. Due to the transfer of 13 study participants to another department, the lost to follow up took place (Fig. 1). Among these transferred 13 nurses, ten did not attend any births per month while two attended 1 to 5 births and one attended more than 50 births per month. Regarding resuscitation, five of these nurses had used a bag and mask to resuscitate a baby while 8 had not. Six of them were staff nurses while seven of them were nurses with a qualification of Bachelor and above.

Among the total of 331 skill drills, 228 (68.9%) drills were conducted at a ventilation rate of 40–60 breathes per minute (adequate ventilation rate). Those who performed 1–5 sessions, 70.4% of them were adequate; those who performed 6–10 sessions, 65.7% of them were adequate; those who performed 11 sessions or more, 70.2% were adequate. There was no correlation of the adequate ventilation with skill drill category (*p* = 0.88) and the level of skill performed (*p* = 0.28) (Table 3).

**Table 3:** Correlation of ventilation rate (40–60 breaths per minute) with drills (*n* = 331)

Variables	Yes (*n* = 228, 68.9%)	No (*n* = 103, 31.1%)	Total (*n* = 331)	*p* value
Drills category				
1–5 sessions	50 (70.4%)	21 (29.6%)	71 (100%)	*p* = 0.28^a^
6–10 sessions	65 (65.7%)	34 (34.3%)	99 (100%)	
≥ 11 sessions	113 (70.2%)	48 (29.8%)	161 (100%)	
Level of skill				
Easy	61 (67.0%)	30 (33.0%)	91 (100%)	*p* = 0.46^a^
Medium	64 (64.6%)	35 (35.4%)	99 (100%)	
Hard	103 (73.1%)	38 (26.9%)	141 (100%)	

^a^Pearson’s Correlation

Out of 47 participants performing the skill drills, 74.5% of them had done effective ventilation with a mean average of 8 skill drills (SD ± 4.78). The remaining 25.5% of them had not done effective ventilation. The mean average skill drills conducted by these participants was done 4 skill drills (SD ± 2.61). With the non-parametric test (Mann Whitney) we found as correlation between number of drills performed by each participant with effective ventilation. An average of 8 drills per participant was associated with effective ventilation in comparison with effective ventilation (*p*-value- 0.032) (Table 4).

**Table 4:** Comparison of total drills with effective ventilation (*n* = 47)

		Mean ± SD	Median (IQR)	*P* value
Effective ventilation	*N* = 16	4.94 ± 3.87	6 (1–17)	0.014^a^
Ineffective ventilation	*N* = 31	8.13 ± 4.58		

^a^Mann Whitney test

## Discussion

This evaluation suggests that study participants who had an average skill drills of 8 in 3 months had effective ventilation (ventilating at the rate of 40–60 breathes per minute). At least 8 skill drills are required to maintain the skills for a period of 3 months for neonatal resuscitation. The number of drills and the level of skill performed has no correlation with the with adequacy of ventilation.

There are several limitations in this study. First, this was an observational study, so equal proportion of drills for each participant would have improved the quality of evidence. Second, this study is done over a period of 3 months, so the waning effect of neonatal resuscitation skills between the drills over a period of time cannot be estimated. Third, there is an observation bias, since the research officers observed the drills.

A study in the US on neonatal resuscitation skill decay following training and with retraining at different time points, showed that the skill retained until 2 months after last training but declined after 4 months [29]. Similar findings were seen in the implementation of HBB in Ghana, where the skill improved following refresher training at 4 months [19]. If the refresher training is provided at periodic intervals, the retention of the pediatric and neonatal intubation skills after simulation-based training is high [30]. Simulation, as a method in the neonatal resuscitation training, improves confidence, knowledge and performance over time in comparison with lectures [31]. However, the skills decay in the simulation group at 6 months [32]. A randomized controlled trial on neonatal resuscitation program on the manikin simulator showed better confidence and satisfaction with high-fidelity manikin [32]. There is a knowledge gap on the optimal dose of the daily skill drills in a high-fidelity simulator for retaining the neonatal resuscitation skills and some studies have demonstrated that simulated drills have an effect on neonatal resuscitation [18, 33–35]. Based on those studies, HBB 2.0 recommends daily drills, however, this recommendation requires better evidence for implementation and advocacy.

## Conclusion

Further reduction in intrapartum-related death requires effective implementation of neonatal resuscitation guideline. The 2015 ILCOR education for improving good clinical practice recommends use of high-fidelity manikin, users’ feedback and periodic training [36]. We demonstrated the optimal dose of simulated drills and feedback required to maintain the neonatal resuscitation skills in simulated settings. Further, evaluation of the simulated drills and feedback in a scale-up setting will provide evidence for generalizability of the result. Further evidence is required, such as optimal dose of skill drills, to have an effect of neonatal resuscitation in a clinical setting.

## Supplementary information

**Additional file 1.** Pictorial form of simulated drills.

**Additional file 2.** Quality Improvement of Perinatal Care (Quality Improvement Tools).

## Data Availability

The datasets used and/or analysed during the current study are available from the corresponding author on reasonable request.

## Abbreviations

CPAP: Continuous Positive Airway Pressure
HBB: Helping Babies Breathe
ILCOR: International Liaison Committee on Resuscitation
OSCE: Objective Structured Clinical Evaluation
QI: Quality Improvement
SDG: Sustainable Development Goal
