# Supplementary figures and images for: Effect of skill drills on neonatal ventilation performance in a simulated setting- observation study in Nepal
Source: BMC Pediatr. 2019 Oct 28;19:387. doi: 10.1186/s12887-019-1723-0 (PMC6816148; doi:10.1186/s12887-019-1723-0)

Supplementary materials

Pictorial form of simulated drills


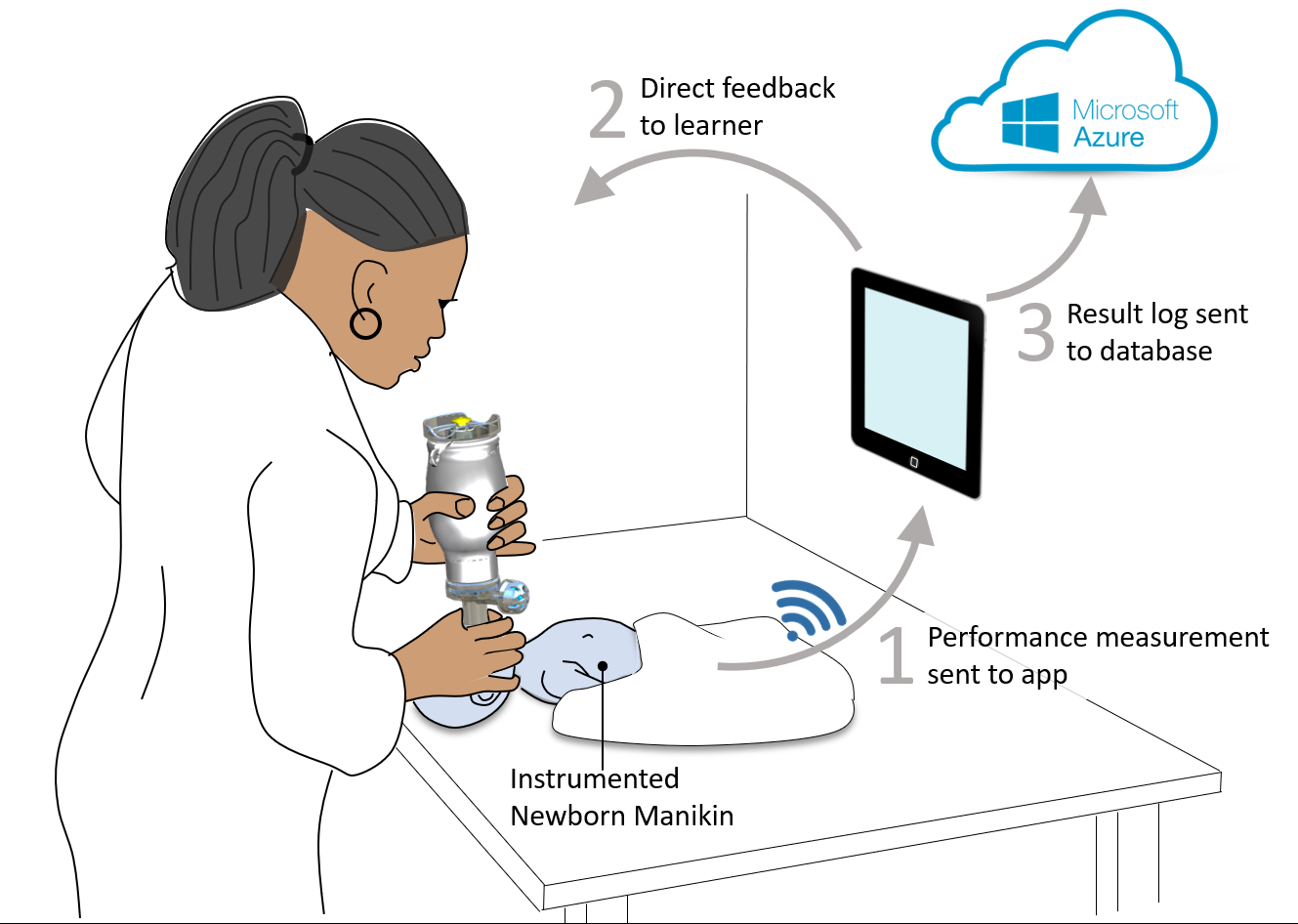

Supplement: Supplementary file 1 — Additional file 1. Pictorial form of simulated drills. [file 12887_2019_1723_MOESM1_ESM.docx]
