# Supplementary material for: Effect of skill drills on neonatal ventilation performance in a simulated setting- observation study in Nepal
Source: BMC Pediatr. 2019 Oct 28;19:387. doi: 10.1186/s12887-019-1723-0 (PMC6816148; doi:10.1186/s12887-019-1723-0)
Supplement: Supplementary file 2 — Additional file 2. Quality Improvement of Perinatal Care (Quality Improvement Tools). [file 12887_2019_1723_MOESM2_ESM.pdf]

Quality Improvement of Perinatal Care (Quality Improvement Tools)

Name of the Hospital:

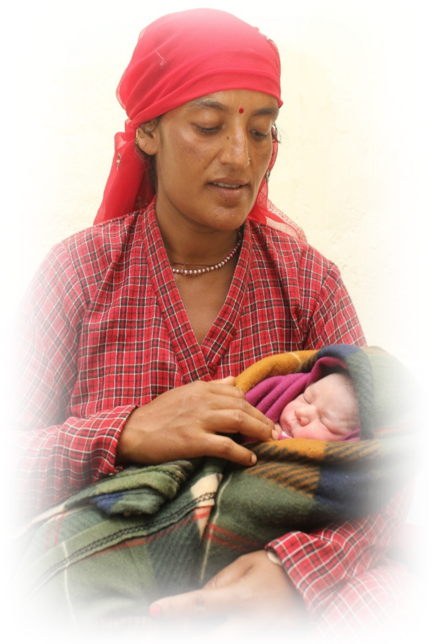

Government of Nepal
Ministry of Health
Department of Health Services
Child Health Division

Table of Contents

SN.	Contents	Page No.
1.	Tool for hospital assessment of readiness and availability of perinatal care services	1
2	Perinatal Care Progress Boards	18
	2.1. Perinatal Care Progress Board in delivery room	18
	2.2. Perinatal Care Progress Board in KMC unit	20
	2.3. Perinatal Care Progress Board in SNCU/NICU	22
	2.4. Perinatal Care Progress Board in Matron's room	24
	2.5. Perinatal Care Progress Board in Medical Superintendent's room	26
3.	Self-assessment checklist	30
	3.1. Self-assessment checklist on Bag and mask skill check on the mannequin	30
	3.2. Self-assessment checklist for preparation for resuscitation before every delivery	31
	3.3. Self-assessment checklist for babies requiring routine immediate newborn care	32
	3.4. Self-assessment checklist for babies requiring ventilation	33
	3.5. Self-assessment checklist for babies who were provided Kangaroo Mother Care	34
	3.6. Self-assessment checklist for babies who were managed for neonatal infection	35

1. Tool for Hospital Assessment of Readiness and Availability of Perinatal Care Services

The purpose of this assessment tool is to evaluate the current capacity of the health facility in providing delivery and newborn care services. This survey elicits information on the status of services, human resources, infrastructure and available equipment. The objective is to summarize the existing resources, identify gaps to inform newborn corner, assist in planning the stabilization unit or special newborn care unit.

The assessment tool consists of six main sections: ***general, availability of service, human resources, infrastructure, equipment and supplies, health information systems and governance and financing***. Each section should be filled in by the Facilitator(s) with the help of Mentor using direct observation, interview of relevant personnel and/or register and schedule checking. The physical observation should be done by both the Mentor and the Facilitator(s) to ensure validity of results. The correct results are to be circled or appropriate numbers and details entered.

PROFORMA FOR FACILITY ASSESSMENT			
SECTION 1: FACILITY IDENTIFICATION INFORMATION			
SN.	Question	Response	Remarks
101.	Date of Assessment (dd/mm/yyyy)	| | | | | | | | |	
102.	Name of District	…………………………………………..	
103.	Type of Hospital	Regional Hospital ………………..……1<br>Sub-Regional hospital ……………..….2<br>Zonal Hospital ………………….…..…...3<br>District Hospital ………………......…...4<br>Other (specify) …………………..………..	
104.	Name of Hospital	…………………………………………..	

SECTION 2: AVAILABILITY OF SERVICES (observe/check)				
Please circle the correct response and fill in the missing information. In addition, please state the source of the information that helped inform the response and whether it is direct observation or an interview with relevant personnel. Annex A contains specific definitions of the services provided for clarification.				
**SN.**	**Question**	**Response**		**Remarks**
**Delivery of Services**		**YES**	**NO**	
**201.**	Is there a 24-hour delivery service?	1	2	
**202.**	**List of Newborn Care Services**			
a)	Immediate newborn care services	1	2	
b)	Resuscitation services	1	2	
c)	Kangaroo Mother Care (KMC)	1	2	
d)	Breastfeeding support	1	2	
e)	Sick Newborn Care Services	1	2	
f)	Others (specify)……………………………….	1	2	
**203.**	**List of Delivery Services**			
a)	Normal deliveries	1	2	
b)	Assisted (Forceps delivery/Vacuum delivery)	1	2	
c)	Manual removal of placenta	1	2	
d)	Administration of placenta oxytocics	1	2	
e)	Administration of magnesium sulphate injections	1	2	
f)	Management of PPH	1	2	
g)	Management of other complications	1	2	
h)	Caesarean section (CS)	1	2	

204.	Referral Services			
a)	Provision of referrals	1	2	If No, go to e)
b)	If yes, name of the referral facility	……………………………….		
c)	What kind of information about the patient is given to the referring hospital? (multiple choice)	Written report……………..1<br>Over phone………………..2<br>None……………………….3<br>Others (specify)…………….		
d)	How would you describe the referral information given?	……………………………….		
e)	Are patients referred to this hospital?	1	2	If No, go to 205
f)	If Yes, name of the facilities that refer patients? (hospitals/health facilities)	……………………………….		
g)	Is there a referral document available for patients that are referred to this hospital?	1	2	If No, go to 205
h)	If Yes, what kind of documentation is available? (if it differs per patient, describe all methods)	……………………………….		
i)	How would you describe the overall referral documentation? (comprehensive or limited)	……………………………….		

205.	Staffing of services	YES	NO	
a)	Is the duty roster (24-hour clock) prepared? (check for availability)	1	2	If No, go to c)
b)	If Yes, are the staff available on their designated shifts?	1	2	
c)	Is there a skilled person in conducting deliveries present at the hospital or on call round the clock, including weekends and public holidays to provide delivery care?	1	2	
d)	Who responds first to complicated deliveries at the hospital? (list all the professionals involved)	…………………………………….		
e)	Is an Obstetrician available to respond to complicated deliveries 24 hours a day?	1	2	
f)	Is a Pediatrician available to respond to complicated deliveries 24 hours a day?	1	2	

206.	Services available in the hospital	YES	NO	
a)	Antenatal Care (ANC) services	1	2	
b)	Postnatal Care (PNC) services	1	2	
c)	Immunization services	1	2	
d)	Family Planning (FP) services	1	2	
e)	Reproductive Health services (RTI/STI)	1	2	
f)	Laboratory services (24-hour)	1	2	
g)	Blood transfusion services	1	2	

SECTION 3: HUMAN RESOURCES				
301.	DESIGNATION	Sanctioned	Fulfilled	Remarks
a)	Medical Superintendent			
b)	Physician			
c)	Neonatologist			
d)	Pediatrician			
e)	Gynecologist/ Obstetrician			
f)	Maternity Ward			
g)	Matron			
h)	Medical Officer			
i)	Ward In-charge			
j)	Staff Nurse			
k)	Auxiliary Nurse Midwives (ANM)			
l)	Health Assistants (HA)			
m)	Support staffs			
n)	SNCU/NICU			
o)	Neonatologist			
p)	Pediatrician			
q)	Ward In-charge			
r)	Staff Nurse			
s)	ANM			
t)	Support staffs			
u)	Others (specify)……………………………….			

302.	TRAINING			
303.	**SBA Training**	**ELIGIBLE**	**RECEIVED**	
a)	Matron			
b)	Medical Officer			
c)	Nursing Supervisor/In-charge			
d)	Staff Nurse			
e)	ANM			
**TOTAL**				
304.	**IMNCI Training**	**ELIGIBLE**	**RECEIVED**	
a)	Medical Superintendent			
b)	Physician			
c)	Neonatologist/Pediatrician			
d)	Gynecologist/Obstetrician			
e)	Matron			
f)	Nursing Supervisor/In-charge			
g)	Staff Nurses			
h)	HA			
i)	ANM			
j)	Others (specify)…………………………….			
**TOTAL**				

305.	Level II Newborn Care Training	ELIGIBLE	RECEIVED	
a)	Medical Superintendent			
b)	Physician			
c)	Neonatologist/Pediatrician			
d)	Gynecologist/Obstetrician			
e)	Matron			
f)	Nursing Supervisor/In-charge			
g)	Staff Nurses			
h)	HA			
i)	ANM			
j)	Others (specify)…………………………….			
**TOTAL**				

SECTION 4: INFRASTRUCTURE, EQUIPMENT AND RECORDS					
The answers to the following questions need to be physically observed by the Facilitator(s) and verified by the Mentor. If the item cannot be observed or the information cannot be gained through observation, the source of the information needs to be stated. This includes the position and relevance of the person informing, website or other informing source.					
**SN.**	**Question**	**Response**			**Remarks**
**401.**	**POWER SUPPLY**				
a)	Is there an electric supply?	YES<br>1	NO<br>2	IRREGULAR<br>3	If No, go to 402
b)	If Yes, check if the electricity is running	YES<br>1	NO<br>2		
c)	Is there lighting in the labor room?	YES<br>1	NO<br>2		If No, go to e)
d)	If Yes, how is the lighting in the labor room?	Strong<br>1	Weak<br>2		
e)	Is there lighting in the SNCU/NICU?	YES<br>1	NO<br>2		If No, go to g)
f)	If Yes, how is the lighting in the SNCU/NICU?	Strong<br>1	Weak<br>2		
g)	Does the hospital have a backup or standby generator for electricity supply?	YES<br>1	NO<br>2		If No, go to 402
h)	If Yes, is the backup or generator functioning? (Accept reported response and state source)	1	2		If No, go to 402
i)	If Yes, is fuel available to run the generator? (Accept reported response and state source)	1	2		

402.	WATER			
a)	What is the main source of water for the hospital?	Piped water……………………….1<br>Tube well or borehole……………..2<br>Dug well……………………………3<br>Tank water supply………………… 4<br>Others (specify)…………………….		
b)	Is the water safe to drink?	YES	NO	If No, go to 403
		1	2	
c)	If Yes, what do you do to make it safe to drink?	Boiling………………………………1<br>Bleaching/chlorination……………..2<br>Strain through a cloth………...……3<br>Water filter…………………………4<br>Sedimentation……………………..5<br>RO Filter……………………………6<br>Others (specify)……………………..		
d)	Is the water outlet (water tap) within 500 meters of the hospital?	YES	NO	
		1	2	
e)	Is the water supply working today?	1	2	

403.	REFERRAL SYSTEM			
a)	Does the hospital have a functional ambulance or other vehicle on-site for referrals?	YES	NO	If No, go to 404
		1	2	
b)	Does the ambulance have fuel in it right now? (check/observe)	1	2	
c)	Does the hospital have a mechanism or system to make phone calls that is available all the time? (mobile phones, landlines)	1	2	If No, go to 404
d)	If Yes, is it available on-site? (check/observe)	1	2	

404.	INFRASTRUCTURE: CAPACITY AND SPACE (check/observe)				
405.	Number of beds in maternity ward	☐☐☐			
406.	Number of beds in labor room	☐☐☐			
407.	Number of beds in postnatal ward	☐☐☐			
408.	Number of beds only for neonates in hospital	☐☐☐			
409.	Number of beds in SNCU/NICU	☐☐☐			
**410.**	**Does the facility have a designated area for the following functions?**	**YES**	**NO**	**If Yes, no. of beds**	
a)	Labor Room	1	2		
b)	Operating Theatre (OT)	1	2		
c)	Postnatal Ward	1	2		
d)	Newborn Corner	1	2		
e)	KMC Corner/Unit	1	2		
f)	Special Care Unit (SNCU/NICU)	1	2		
g)	Breastfeeding corner (for patients and staffs both)	1	2		
h)	Handwashing space (labor ward)	1	2		
i)	Handwashing space (SNCU/NICU)	1	2		
j)	Boiling and autoclaving	1	2		
k)	Laundry	1	2		

l)	Clean utility room (for storing used and contaminated materials)	1	2		
m)	Soiled utility room (for storing used and contaminated materials)	1	2		
n)	Store	1	2		
o)	Laboratory Room	1	2		
p)	Doctor's Room	1	2		
q)	Nurses Room	1	2		
411.	In the last 3 months, have any in-patients in the maternity ward shared beds at any time before or after delivery?	YES<br>1	NO<br>2		
412.	In the last 3 months, have any in-patients in the maternity ward slept on the floor?	YES<br>1	NO<br>2		
413.	In the last 3 months, have the maternity patients delivered on the floor in a corridor or bathroom due to lack of beds?	YES<br>1	NO<br>2		
414.	Right now, are there any empty beds? (Maternity Ward only)	YES<br>1	NO<br>2		
**415.**	**LOCATION OF EQUIPMENT/SUPPLIES (check/observe)**				
a)	Is the delivery set located in the delivery ward?	1	2		
b)	Where is the resuscitation equipment located? (multiple response)	Admission ward……………………1<br>Labor ward………………………...2<br>PNC ward…………………………..3<br>OT…………………………………..4<br>SNCU/NICU……………………… 5<br>Others (specify)……………………..			

EQUIPMENT AND SUPPLIES

The purpose of this listing is to have an inventory of all available equipment at the facility. Availability refers to whether the item is in fact present and can be physically observed. The functionality refers to the condition of the supplies, whether the item is in working order. Function means it is in working condition and packaged implies it is sealed and unopened.

Available---1; Not available---2; Functional---3; Non-functional---4; Packaged---5; Unable to determine ---6

SN.	Monitoring Equipment	Availability	Functionality	Remarks
a)	Stethoscope with neonatal chest piece	1 2	3 4 5 6	
b)	Non-invasive BP monitors	1 2	3 4 5 6	
c)	Heart rate/apnea monitor	1 2	3 4 5 6	
d)	Pulse oximeter	1 2	3 4 5 6	
e)	Low reading clinical thermometers	1 2	3 4 5 6	
f)	Room thermometers	1 2	3 4 5 6	
g)	Electronic baby weighing scale	1 2	3 4 5 6	
h)	Mechanical baby weighing scale	1 2	3 4 5 6	
**416.**	**Equipment for Management**			
a)	Radiant warmer	1 2	3 4 5 6	
b)	Phototherapy unit	1 2	3 4 5 6	
c)	CPAP	1 2	3 4 5 6	
d)	Ventilator	1 2	3 4 5 6	
e)	Glucometer	1 2	3 4 5 6	
f)	Others (specify)…………………..…...	1 2	3 4 5 6	

**417.**	**Resuscitation Equipment**			
a)	Self-inflating bags	1 2	3 4 5 6	
b)	Foot operated suction pumps/mucus trap	1 2	3 4 5 6	
c)	Penguin suction	1 2	3 4 5 6	
d)	Bag and Mask	1 2	3 4 5 6	
**418.**	**Oxygenation Facility**			
a)	Centralized	1 2	3 4 5 6	
b)	Oxygen cylinder	1 2	3 4 5 6	
c)	Concentrator	1 2	3 4 5 6	
d)	Head boxes for delivery of oxygen	1 2	3 4 5 6	
**419.**	**Equipment for Investigation**			
a)	Micro-hematocrit (Hemoglobinometer)	1 2	3 4 5 6	
b)	Dextrometer	1 2	3 4 5 6	
c)	Microscope	1 2	3 4 5 6	
d)	Total Serum Bilirubin	1 2	3 4 5 6	
e)	HbsAg	1 2	3 4 5 6	
f)	HIV	1 2	3 4 5 6	
g)	Blood Grouping	1 2	3 4 5 6	
h)	Total Blood Count	1 2	3 4 5 6	
i)	C-Reactive Protein (C-RP)	1 2	3 4 5 6	
j)	Blood Glucose	1 2	3 4 5 6	

k)	Lumbar puncture	1 2	3 4 5 6	
l)	Renal Function Test (RFT)	1 2	3 4 5 6	
m)	Blood Culture	1 2	3 4 5 6	
n)	Urine Culture	1 2	3 4 5 6	
o)	CSF Culture	1 2	3 4 5 6	
p)	X-Ray	1 2	3 4 5 6	
**420.**	**General Equipment**			
a)	Generator	1 2	3 4 5 6	
b)	Invertors	1 2	3 4 5 6	
c)	Washing Machine	1 2	3 4 5 6	
d)	Refrigerator	1 2	3 4 5 6	
e)	Computer	1 2	3 4 5 6	
f)	Wall clock (with ‘seconds’ hand)	1 2	3 4 5 6	
g)	Surgical instruments	1 2	3 4 5 6	
h)	Spot lamps	1 2	3 4 5 6	
i)	Air Conditioner	1 2	3 4 5 6	
j)	Autoclave equipment	1 2	3 4 5 6	

SECTION 5: HEALTH INFORMATION SYSTEMS				
**SN.**	**Question**	**Response**		**Remarks**
		**YES**	**NO**	
**501.**	**INPUT**			
502.	Does the facility carry out audits or case reviews or maternal deaths on a routine basis?	1	2	
503.	Does the facility carry out audits or case reviews or newborn deaths/stillbirths on a routine basis?	1	2	
504.	Does this hospital have a system in place to regularly collect MNH service data?	1	2	
**505.**	**OUTPUT**			
506.	Does this facility regularly complete any reports containing MNH services?	1	2	If No, go to 508
507.	If Yes, who does it?	………………………………………..		
508.	What is the main source of the data for the reports?	………………………………………..		
509.	How frequently are these reports compiled?	………………………………………..		
**510.**	**DISSEMINATION**			
511.	Who has the access to the compiled data? (multiple choice)	All staffs…………………………..….1<br>Decision makers…………………..…2<br>Public………………………………..….3<br>NGOs/INGOs……………………..…..4<br>No one………………………………....5<br>Others (specify)……………………		

512.	Is the data displayed	1	2	If No, go to Section 6
513.	If Yes, where is it displayed?	…………………………………………..		
514.	In what format is the data presented? (aggregated/graphs) Confirm with the medical recorder/stat officer	…………………………………………..		

SECTION 6: GOVERNANCE AND FINANCING				
**SN.**	**Question**	**Response**		**Remarks**
601.	Is there a hospital governance committee?	YES	NO	If No, go to 605
		1	2	
602.	If Yes, how frequently do they meet? (times/year)	☐☐		
603.	What is the number of work/action plans created?	☐☐		
604.	How many work/action plans have been reviewed?	☐☐		
605.	Is there a hospital QI/MPDR committee?	YES	NO	If No, go to 609
		1	2	
606.	If Yes, how frequently do they meet? (times/year)	☐☐		
607.	What is the number of work/action plans created?	☐☐		
608.	How many work/action plans have been reviewed?	☐☐		

609.	How is the hospital financed?	Government of Nepal…………….1<br>Hospital Development Committee…………………………..2<br>NGOs/INGOs………………………3<br>Private funders……………………4<br>Others (specify)……………………	
610.	What is the main source of revenue generated? (i.e. laboratory tests, admission fee, etc.)<br>(mention all the sources)	………………………………………	

Facilitator's Name & Signature………………………………………

Mentor's Name & Signature…………………………………………

Date………………………..

2.1. Perinatal Care Progress Board in Delivery Room

Indicators	Month: Week:							Weekly compilation (previous week)
	Days							
	Sun	Mon	Tue	Wed	Thu	Fri	Sat	
Total birth								
Total Still birth								
Number of babies with Low Birth Weight								
Number of babies who did not cry at birth								
Number of babies who are successfully resuscitated with bag & mask								
Number of babies who were resuscitated with bag & mask within 1 minute								
Number of babies who were initiated breastfeeding within 1 hour of birth								

Instructions for use

1. This progress board will be placed visibly in the delivery room.
2. This progress should be maintained on a daily basis by the facilitator or a person assigned by the facilitator. The number in the row for respective indicator should be filled in the morning hours of following day. For example, the facilitator/ health worker will fill the number of respective indicator of Sunday in the morning of Monday.
3. At the end of the week the facilitator should compile the numbers and note in a diary.
4. After completion of the week, the numbers should be erased and the process should be started for the next week.
5. Description of indicators

Total birth	Write the total number of births (live and still birth in a day).
Total Still birth	Write the total number of still births.
Number of babies with Low Birth Weight	Write the total number of babies who were born with low birth weight.
Number of babies who did not cry at birth	Write the total number of babies who did not cry at birth
Number of babies who are successfully resuscitated with bag & mask	Write the total number of babies who were successfully resuscitated with bag and mask
Number of babies who were resuscitated with bag & mask within 1 minute	Write the total number of babies who were resuscitated with bag & mask within 1 minute
Number of babies who were initiated breastfeeding within 1 hour of birth	Write the total number babies who were initiated breastfeeding within 1 hour of birth

2.2. Perinatal Care Progress Board in KMC Unit

Indicators	Month:			Week:				Weekly compilation (previous week)
	Days							
	Sun	Mon	Tue	Wed	Thu	Fri	Sat	
Number of preterm babies admitted to KMC								
Number of LBW babies (<2000g) admitted to KMC								
Number of LBW newborns receiving near continuous KMC								
Number of LBW newborns receiving breast milk feeding								
Number of babies referred to SNCU/NICU								

Instructions for use

1. This progress board will be placed visibly in the KMC unit.
2. This progress should be maintained on a daily basis by the facilitator or a person assigned by the facilitator. The number in the row for respective indicator should be filled in the morning hours of following day. For example, the facilitator/ health worker will fill the number of respective indicator of Sunday in the morning of Monday.
3. At the end of the week the facilitator should compile the numbers and note in a diary.
4. After completion of the week, the numbers should be erased and the process should be started for the next week.
5. Description of indicators

Number of preterm babies admitted to KMC unit	Write the total number of preterm babies admitted to KMC unit.
Number of LBW babies (<2000g) admitted to KMC	Write the total number LBW babies with < 2000g weight admitted to KMC unit.
Number of LBW newborns receiving near continuous KMC	Write the total number of LBW babies who have received KMC service with permitted intervals as per protocol.
Number of LBW newborns receiving breast milk feeding	Write the total number of LBW newborns who is continuously receiving breast milk feeding
Number of babies referred to NICU/SNCU	Write the total number babies under KMC who were referred to NICU/SNCU due to complications.

2.3. Perinatal Care Progress Board in Sick Newborn Care Unit (NICU/SNCU)

**Indicators**	**Month:**				**Week:**			**Weekly compilatio (previous week)**
	**Days**							
	**Sun**	**Mon**	**Tue**	**Wed**	**Thu**	**Fri**	**Sat**	
Number of newborns admitted								
Number of inborn babies admitted								
Number of babies who had Jaundice								
Number of babies who had Respiratory Distress Syndrome (RDS)								
Number of babies with sepsis who received antibiotics								
Number of babies who had Hypoxic Ischemic Encephalopathy (HIE)								
Number of babies who received CPAP								
Number of babies who received ventilation								
Number of first day neonatal deaths								
Number of early neonatal deaths (0 to 6 days)								

Instructions for use

1. This progress board will be placed visibly in the SNCU/NICU.
2. This progress should be maintained on a daily basis by the facilitator or a person assigned by the facilitator. The number in the row for respective indicator should be filled in the morning hours of following day. For example, the facilitator/ health worker will fill the number of respective indicator of Sunday in the morning of Monday.
3. At the end of the week the facilitator should compile the numbers and note in a diary.
4. After completion of the week, the numbers should be erased and the process should be started for the next week.
5. Description of indicators

Number of newborns admitted	Write the total number of newborns (out-born and inborn) admitted to SNCU/NICU
Number of inborn babies admitted	Write the total number of inborn babies admitted in SNCU/NICU
Number of babies who had Jaundice	Write the total number of admitted babies who had Jaundice
Number of babies who had Respiratory Distress Syndrome (RDS)	Write the total number of admitted babies who had Respiratory Distress Syndrome
Number of babies with sepsis who received antibiotics	Write the total number of babies admitted in SNCU/NICU who received antibiotics
Number of babies who had Hypoxic Ischemic Encephalopathy (HIE)	Write the total number of babies admitted in SNCU/NICU who had Hypoxic Ischemic Encephalopathy (HIE)
Number of babies who received CPAP	Write the total number of babies admitted in SNCU/NICU who received CPAP
Number of babies who received ventilation	Write the number of babies who received ventilation support
Number of first day neonatal deaths	Write the total number of newborns who died during the first 24 hours of birth during a month
Number of early neonatal deaths (0 to 6 days)	Write the total number of newborns who died during first and 6th day of life

2.4. Perinatal Care Progress Board in Progress board in Matron's room

Indicators	Month:				
	Week 1	Week 2	Week 3	Week 4	Week 5
Total birth					
Total Still birth					
Number of babies with Low Birth Weight					
Number of babies who did not cry at birth					
Number of babies who are successfully resuscitated with bag & mask					
Number of babies who were resuscitated with bag & mask within 1 minute					
Number of babies admitted to KMC					
Number of LBW newborns receiving near continuous KMC					
Number of babies with sepsis who received antibiotics					
Number of babies who received CPAP					
Number of babies who receive ventilation					
Number of first day neonatal deaths					
Number of early neonatal deaths (0 to 6 days)					

Instructions for use

1. This progress board will be placed visibly in the Matron's room.
2. This progress should be maintained on a weekly basis by the facilitator or a person assigned by the facilitator. The number in the row for respective indicator should be filled after the completion of a week. The facilitator/ health worker will fill the number of respective indicator of previous week on the morning hours of Sunday.
3. At the end of the month the facilitator should compile the numbers and note in a diary.
4. After completion of the month, the numbers should be erased and the process should be started for the next month.
5. Description of indicators: The numbers for each indicator should be derived from the progress boards being used in Delivery Unit, KMC Unit/Corner and SNCU/NICU.

2.5. Progress board in in the Medical Superintendent's room

Indicators	Month											
	1	2	3	4	5	6	7	8	9	10	11	12
Stillbirth Rate												
Proportion of babies who had Low Birth Weight												
Proportion of babies who did not cry at birth												
Proportion of non-crying babies who are successfully resuscitated with bag & mask												
Proportion of non-crying babies who were resuscitated with bag & mask within 1 minute												
Number of babies admitted to KMC												
Proportion of LBW newborns receiving near continuous KMC												
Proportion of babies with sepsis who received antibiotics												
First day neonatal mortality rate												
Early neonatal (0 to 6 days) mortality rate												

Instructions for use

1. This progress board will be placed visibly in the room of Medical Superintendent.
2. This progress should be maintained on a monthly basis by the medical record officer/medical recorder. The number in the row for respective indicator should be filled after the completion of each month.
3. At the end of the month the facilitator should compile the numbers and note in a diary.
4. After completion of the month, the numbers should be erased and the process should be started for the next month.
5. Description of indicators

Stillbirth Rate	**Numerator:** Total number of still births in a month<br>**Denominator:** Total births during the same month<br>**Multiplier:** 1000
Proportion of babies who had Low Birth Weight	**Numerator:** Total number babies with low birth weight (<2500 g) in a month<br>**Denominator:** Total live births during the same month<br>**Multiplier:** 100
Proportion of babies who did not cry at birth	**Numerator:** Total number of babies who did not cry at birth in a month<br>**Denominator:** Total births during the same month<br>**Multiplier:** 100
Proportion of non-crying babies who are successfully resuscitated with bag & mask	**Numerator:** Total number of non-crying babies who are successfully resuscitated with bag & mask<br>**Denominator:** Total number of non-crying babies who were resuscitated with bag & mask<br>**Multiplier:** 100

Proportion of non-crying babies who were resuscitated with bag & mask within 1 minute	**Numerator:** Total number of non-crying babies who were resuscitated with bag & mask within 1 minute<br>**Denominator:** Total number of babies who did not cry at birth<br>**Multiplier:** 100
Number of babies admitted to KMC	Write the total number of babies admitted to KMC unit during a month
Proportion of LBW newborns receiving near continuous KMC	**Numerator:** Total number of babies receiving near continuous KMC during a month<br>**Denominator:** Total number of LBW babies<br>**Multiplier:** 100
Proportion of babies with sepsis who received antibiotics	**Numerator:** Total number of babies with sepsis who received antibiotics<br>**Denominator:** Total number of babies with sepsis admitted to SNCU/NICU<br>**Multiplier:** 100
First day neonatal mortality rate	**Numerator:** Total number of newborns who died during the first 24 hours of birth during a month<br>**Denominator:** Total live births<br>**Multiplier:** 1000
Early neonatal (0 to 6 days) mortality rate	**Numerator:** The total number of newborns who died during first and sixth day of birth during a month.<br>**Denominator:** Total live births during a month<br>**Multiplier:** 1000

Summary of Checklist for Perinatal Care in Hospital

Name of Checklist	Type of checklist	Objective	How	Who	Where	when	How is it going to be used
**Bag-and-mask skill check**	Simulated	To improve the simulated skill on neonatal resuscitation on a mannequin	Bag-and-mask skill check in a mannequin (self and peer)<br>Recording of the steps followed	Health worker	Labor room or hand over room	At the beginning of taking handover every day on duty	
**Preparation for resuscitation before every delivery**	Clinical	To improve the clinical performance on preparation for resuscitation before every birth	Preparation for the materials for resuscitation before each delivery in the resuscitation corner	Health worker	Resuscitation corner	During labor, just before delivery	
**Immediate routine newborn care**	Clinical	To improve the clinical performance on immediate routine newborn care	Immediate routine newborn care	Health worker	Labor room	Immediately after delivery	
**Care of the babies who were resuscitated**	Clinical	To improve the clinical performance on resuscitation	Neonatal resuscitation within 1 minute	Health worker	Labor room	Immediately after delivery	
**Care of the babies who are born small**	Clinical	To improve the clinical performance on care of small babies	KMC for babies who are born small	Health worker	KMC corner or Unit	After provision of KMC counselling	
**Management of the babies who have neonatal sepsis**	Clinical	To improve the clinical performance on management of neonatal sepsis	Management of neonatal sepsis	Health worker	SNCU/NICU	During or after management of neonatal sepsis	

3.1. Self-assessment checklist on Bag-and-Mask skill check on the mannequin

Instruction - On a daily basis, before taking the hand over for the duty, the health worker need to do a bag-and-mask skill check on the mannequin. After the bag-and-mask skill check the health workers need to fill in the self-assessment checklist.

After filling up the self-assessment checklist, the health workers need to tear the filled in paper and put/clip it on a separate index form or area.

Name of the hospital ________________________________

Name of health worker ___________________________________

Date (dd/mm/yyyy) ____________________ **Time (hh:mm)** _______________________

Scoring keys: Y=Yes, N=No, NA= Not applicable **Score: All Yes =1, Any No =0**

Performance area	Checklist	Y/N	NA
**1. Check equipment and select correct mask**	Test the function of Bag and Mask		
	Make sure mask fits the mannequin's face		
**2. Apply the mask to make to a firm seal in the mouth and nose of mannequin**	Extend the head, place the mask on the chin, then over the mouth and nose		
	A firm seal permits chest movement when the bag is squeezed		
**3. Ventilate 40 breaths per minute**	The rate should not be less than 30 or more than 50 breaths per minute		
4. **Look for chest movement**	Check that for every ventilation breath produces chest movement		

Signature of the health worker……………………………………….

3.2. Self-assessment checklist for preparation for resuscitation before every delivery

Instruction - For every birth, the health workers need to prepare for resuscitation. After the delivery of each baby, the health worker need to fill in the self-assessment checklist.

After filling up the self-assessment checklist, the health workers need to tear the filled in paper and put/clip it on a separate index form or area.

Name of the hospital ______________________________

Name of health worker ______________________________

Date (dd/mm/yyyy) ____________________ **Time (hh:mm)** ______________________

Scoring keys: Y=Yes, N=No, NA= Not applicable **Score: All Yes =1, Any No =0**

Steps	Y/N	NA
1. Identification of helper and review of emergency plan with the helper		
2. Prepared the delivery room for delivery		
3. Washed hands		
4. Prepared the area for ventilation (resuscitation corner)		
5. Assembled all supplies and equipment (Gloves, clothes, head covering, scissors, clamps, suction device, Bag-Mask, stethoscope, timer)		
6. Checked the bag and mask for ventilation		

Signature of the health worker…………………………………….

3.3. Self-assessment checklist for babies requiring routine immediate newborn care

Instruction- For every birth, if the baby is breathing well, routine immediate newborn care needs to be provided. After the delivery of each baby, the health worker need to fill in the self-assessment checklist.

After filling up the self-assessment checklist, the health workers need to tear the filled in paper and put/clip it on a separate index form or area.

Name of the hospital ________________________________

Name of health worker ________________________________

Date (dd/mm/yyyy) ____________________ **Time (hh:mm)** ________________________

Scoring keys: Y=Yes, N=No, NA= Not applicable **Score: All Yes =1, Any No =0**

Steps	Y/N	NA
1. Babies dried thoroughly		
2. Checked whether the baby is crying or not		
3. Keep the baby warm (Cover head and body, Position skin to skin with the neck slightly extended)		
4. Breathing of the baby checked		
5. Umbilical cord clamped or tied and cut the cord (within 1 – 3 min after birth)		
6. Reposition baby on mother's chest to encourage breastfeeding within 1 hour of birth		
7. Assess any danger signs and complete the birth record		

Signature of the health worker……………………………………….

3.4. Self-assessment checklist for babies requiring ventilation

Instruction - For babies who required resuscitation, the health worker need to fill in the self-assessment checklist. After filling up the self-assessment checklist, the health workers need to tear the filled in paper and put/clip it on a separate index form or area.

Name of the hospital ___________________________________

Name of health worker ___________________________________

Date (dd/mm/yyyy) ______________________ Time (hh:mm) ______________________

Scoring keys: Y=Yes, N=No, NA= Not applicable **Score: All Yes =1, Any No =0**

Steps	Y/N	NA
1. Babies dried thoroughly		
2. Checked whether the baby is crying or not		
3. If baby is not crying, baby kept warm and airway cleared if needed		
4. Breathing of the baby stimulated (rubbing the back)		
5. Breathing of the baby checked		
6. If the baby is not breathing, Umbilical cord clamped or tied and cut the cord		
7. Moved baby to ventilation area		
8. Stand towards the baby's head and correct mask selected		
9. Ventilation initiated within 1 minute after delivery		
10. Ventilation at 40 breaths per minute		
11. Recognized chest moving/not moving		
12. Recognized breathing		
13. If baby is not breathing well, Call for help		
14. Continued ventilation		
15. Recognized breathing and assessed heart rate		
16. If heart Rate less than 100/minute or normal with no breathing, continue ventilation and refer for advanced care		
17. If breathing well and Heart rate normal		
18. Stopped ventilation, monitored baby		
19. Assessed malformation		

Signature of the health worker……………………………………….

3.5. Self-assessment checklist for babies who were provided Kangaroo Mother Care (KMC)

Instruction - For babies who require KMC, the health worker is requested to fill in the self-assessment checklist. After filling up the self-assessment checklist, the health workers need to tear the filled in paper and put/clip it on a separate index form or area.

Name of the hospital ________________________________

Name of health worker ________________________________

Date (dd/mm/yy)____________________ **Time (hh:mm)** _______________________

Scoring keys: Y=Yes, N=No, NA= Not applicable **Score: All Yes =1, Any No =0**

Steps	Y/N	NA
1. Mother or care-giver counselled on benefits of KMC		
2. privacy for the client ensured		
3. Hand washing done before handling the newborn		
4. Baby dressed in cap, socks, nappy and front open clothes/shirt.		
5. Baby placed in between mother's breasts in upright position		
6. Baby's head turned to one side in slightly extended position		
7. Hips are flexed and abducted and arms are also flexed (frog position)		
8. Baby's abdomen is at the level of the mother's epigastrium		
9. Baby is supported underneath gluts by a sling/binder		
10. Position of head and neck (alternate neck position), airway, breathing, color and temperature monitored		
11. Encourage mother/caregiver to sit in a comfortable position and counsel on the need to provide KMC for 24 hours if possible		

Signature of the health worker……………………………………….

3.6. Self-assessment checklist for babies who were managed for neonatal infection

Instruction- For babies who are managed of neonatal infection, the health worker is requested to fill in this self-assessment checklist. After filling up the self-assessment checklist, the health workers need to tear the filled in paper and put/clip it on a separate index form or area.

Name of the hospital

Name of health worker

Date (dd/mm/yyyy)______________________ Time (hh:mm) __________________________

Scoring keys: Y=Yes, N=No, NA= Not applicable **Score: All Yes =1, Any No =0**

Steps	Y/N	NA
1. Washed hands before handling newborn		
2. Checked the environment and provide warmth, ensured a consistent normal temperature for the baby.		
3. Started intravenous line		
4. Infuse normal saline 10ml/kg over 30 minutes if CRT>3 seconds, repeat the same 1-2 times, if perfusion continues to be poor		
5. Infuse 10% dextrose 2 ml/kg stat		
6. Injected Vitamin K1 mg IM (if birthweight ≥ 1000 gm) and 0.5 mg if birthweight <1000 gm		
7. Started oxygen by hood or mask, if cyanosed or grunting		
8. Provided gentle physical stimulation, if apnoeic. Provided bag and mask ventilation with oxygen if breathing is inadequate		
9. Avoid enteral feed if hemodynamically compromised, give maintenance IV fluids		
10. Considered use of dopamine if perfusion is persistently poor		
11. Counselled mother and family infection prevention practices<br>a. Hand washing<br>b. Minimum visitors<br>c. Clean clothing		
12. Monitored the Clinical Signs of bacterial infection throughout the process and responded accordingly.		

Signature of the health worker……………………………
